# Sustainable Self-Training Pig Detection System with Augmented Single Labeled Target Data for Solving Domain Shift Problem

**DOI:** 10.3390/s25113406

**Published:** 2025-05-28

**Authors:** Junhee Lee, Heechan Chae, Seungwook Son, Jongwoong Seo, Yooil Suh, Jonguk Lee, Yongwha Chung, Daihee Park

**Affiliations:** 1 Info Valley Korea Co., Ltd., Anyang 14067, Republic of Korea; lyjourney@invako.kr (J.L.); chai@invako.kr (H.C.); sso7199@invako.kr (S.S.); yoor0815@invako.kr (Y.S.); 2 Department of Computer Convergence Software, Korea University, Sejong 30019, Republic of Korea; seojongwoong@korea.ac.kr (J.S.); eastwest9@korea.ac.kr (J.L.)

**Keywords:** monitoring system, object detection, domain shift, data augmentation, genetic algorithm, self-training

## Abstract

**Highlights:**

**What are the main findings?**

- The proposed approach combining a single labeled target sample, genetic algorithm-optimized augmentation, and super-low-threshold self-training more than doubled pig detection accuracy under domain shift (36.86 → 90.62), achieving a performance comparable to fully supervised training;
- The proposed system maintained high detection precision across diverse pigsty conditions (e.g., varying lighting, camera viewpoints, and pig sizes), demonstrating robust object detection performance against real-world domain shifts with minimal labeled data.

**What is the implication of the main finding?**

- The proposed solution requires only one labeled target image for model adaptation, drastically reducing the manual labeling effort and enabling quick, cost-effective deployment of pig detection models in new farm environments;
- By ensuring high accuracy under real-world domain shift scenarios, the system enables practical and scalable intelligent livestock management, improving monitoring reliability and overall farm efficiency.

**Abstract:**

As global pork consumption rises, livestock farms increasingly adopt deep learning-based automated monitoring systems for efficient pigsty management. Typically, a system applies a pre-trained model on a source domain to a target domain. However, real pigsty environments differ significantly from existing public datasets regarding lighting conditions, camera angles, and animal density. These discrepancies result in a substantial domain shift, leading to severe performance degradation. Additionally, due to variations in the structure of pigsties, pig breeds, and sizes across farms, it is practically challenging to develop a single generalized model that can be applied to all environments. Overcoming this limitation through large-scale labeling presents considerable burdens in terms of time and cost. To address the degradation issue, this study proposes a self-training-based domain adaptation method that utilizes a single label on target (SLOT) sample from the target domain, a genetic algorithm (GA)-based data augmentation search (DAS) designed explicitly for SLOT data to optimize the augmentation parameters, and a super-low-threshold strategy to include low-confidence-scored pseudo-labels during self-training. The proposed system consists of the following three modules: (1) data collection module; (2) preprocessing module that selects key frames and extracts SLOT data; and (3) domain-adaptive pig detection module that applies DAS to SLOT data to generate optimized augmented data, which are used to train the base model. Then, the trained base model is improved through self-training, where a super-low threshold is applied to filter pseudo-labels. The experimental results show that the proposed system significantly improved the average precision (AP) from 36.86 to 90.62 under domain shift conditions, which achieved a performance close to fully supervised learning while relying solely on SLOT data. The proposed system maintained a robust detection performance across various pig-farming environments and demonstrated stable performance under domain shift conditions, validating its feasibility for real-world applications.

## 1. Introduction

According to the Organization for Economic Co-operation and Development (OECD) and the Food and Agriculture Organization (FAO)’s Agriculture Outlook 2022–2031 [1], global pork consumption is expected to increase to 129 million tons over the next decade. Pork is projected to become the most consumed meat not only in Asia but also in Europe. To meet this continuously growing demand, livestock farms are expanding their production scale by enlarging their facilities [2]. However, expanding facilities or hiring additional staff poses significant challenges for small- and medium-sized farms with limited budgets. Researchers have proposed various pigsty management systems to enhance farm management efficiency [3,4,5].

Information and communication technology (ICT) has been introduced as a monitoring technology for pigsty management due to its ability to collect data and analyze information using various algorithms automatically. Several studies have been conducted in this area. For example, researchers have developed technologies to detect abnormal behaviors early, such as tail-biting for dominance establishment, reduced activity due to health issues, and coughing caused by respiratory diseases. By effectively addressing these abnormal situations, farms can minimize economic losses and improve breeding efficiency [6,7,8,9,10,11,12].

In the early stages of pigsty monitoring system adoption, computer vision techniques were primarily used [9,10,11,12]. Traditional computer vision approaches relied on feature descriptors, such as Scale-Invariant Feature Transform (SIFT) [13], sped-up robust features (SURF) [14], and Binary Robust Independent Elementary Features (BRIEF) [15], combined with machine learning models, like support vector machines (SVMs) and K-Nearest Neighbors (KNN), to detect pigs and analyze their behavior [16]. However, these methods required manual feature extraction, making it challenging to effectively process diverse and unstructured data. Their generalization performance had limits, leading to significant performance degradation in new environments [16,17,18].

Deep learning-based computer vision technologies have recently been actively introduced to overcome these limitations [19,20,21]. Convolutional neural network (CNN)-based models automatically learn features from data and maintain high accuracy across various environments, making them a promising solution to the limitations of traditional methods. Unlike conventional approaches, deep learning models can effectively model complex patterns and interactions, significantly improving the accuracy of abnormal behavior detection and health monitoring [22,23,24]. Deep learning-based monitoring systems have progressed beyond the experimental stage, achieving high accuracy levels that meet industrial demands, making them applicable to real farms [25]. However, technical challenges remain to ensure stable performance in real-world environments, with domain shift being one of the most critical issues.

Domain shift [26,27] is a significant cause of model performance degradation, arising from distribution differences between source and target data. Deep learning models operate based on pre-trained datasets, but discrepancies may exist between training data (source) and monitoring data (target) in real farm environments. Differences between the data collection environment and the new environment can lead to model performance deterioration. For example, variations in lighting conditions, camera angles, pigsty structures, pig growth stages (size changes), breed differences, and other environmental factors can create different data distributions from the environment in which the model was initially trained. The greater the disparity between the source and target data, the more significant the drop in model accuracy [28].

One common approach to addressing this issue is incorporating target data into the training dataset. However, generating large-scale annotated target data is labor-intensive and time-consuming [29]. Consequently, researchers have explored self-training techniques to enable models to automatically learn from unlabeled target data [30,31,32,33].

**Figure 1:**
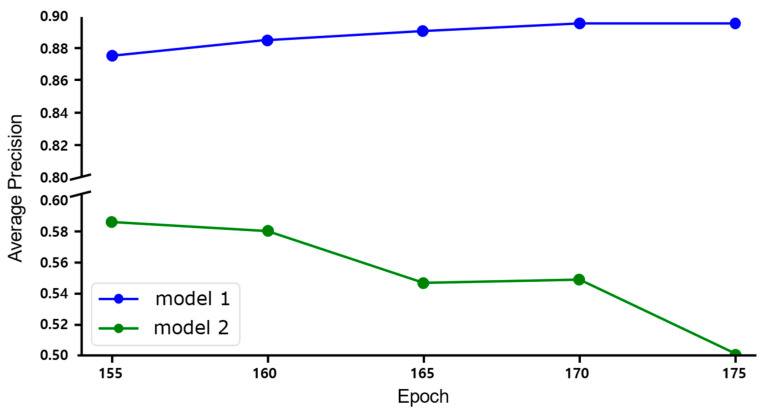
A graph illustrating the contrasting self-training results based on the base model. Although the same data and parameters were applied, the self-training outcomes differed due to the base model’s accuracy variation. The blue-colored Model 1 shows a performance improvement as the training progressed, whereas the green-colored Model 2 exhibits performance degradation.

Self-training involves using a pre-trained model on source data to generate pseudo-labels for unlabeled target data, allowing the model to learn target data features and mitigate domain shift issues. However, since pseudo-labels are generated without human intervention, their accuracy depends on the base model’s performance. If the base model has low accuracy, the generated pseudo-labels may be incorrect, potentially degrading the final model’s performance instead of improving it (see Figure 1). Despite this limitation, existing studies aim to train high-accuracy models by allowing the model to learn target-domain features without any labeled target data [34,35,36]. This approach disregards the most reliable method for improving model accuracy—adding labeled target data. Therefore, this study proposes a method that uses a minimal amount of manually labeled data, called Single Label On Target (SLOT), which refers to utilizing only a single labeled example from the target domain, to train the base model for self-training.

The SLOT data refer to data that have been manually annotated, specifically containing ground truth labels for pig objects in images collected from the target-domain pigsty. Utilizing SLOT data provides the following advantages: (1) Since it includes high-quality annotations for the target domain, it allows for the application of intensive and diverse augmentations. (2) It enables accurate learning of the target domain’s features, including background and structures. Learning is particularly effective in monitoring systems where background and structures remain unchanged once installed. If the target domain has unique structures or patterns, SLOT provides an opportunity to learn these characteristics. (3) As the base model for self-training learns the target domain’s features, pseudo-labels’ reliability improves, minimizing the risk of accuracy degradation in subsequent training stages. As a result, SLOT serves as an efficient approach that maximizes benefits while requiring minimal human labor.

This study proposes a camera-based adaptive pig detection system that addresses the domain shift problem by leveraging SLOT as minimal human-labeled data to train the initial base model for self-training. The proposed system first applies a key frame selection algorithm to remove redundant data from videos collected via sensors in the target domain and selects SLOT data from the remaining frames. Next, the selected SLOT data undergo Data Augmentation Search (DAS), a method designed to systematically find the best augmentation parameters using a Genetic Algorithm (GA), to generate optimally augmented images. These images and the training dataset are then used to train the base model, which is subsequently refined through self-training. During pseudo-label generation, a super-low threshold is applied to include objects with lower confidence scores in the training process, ensuring that a broader range of target-domain data is utilized.

Ultimately, the core novelty of this study lies in proposing a sustainable and detector-agnostic pig detection system that addresses the domain shift problem using only a minimal amount of labeled target data, while enabling generalization across diverse environments with minimal supervision. To demonstrate the system’s versatility and consistency, we conducted experiments using multiple object detection models, thereby validating its applicability and detector independence. The key contributions of this paper are summarized as follows:

1. This study effectively addressed the domain shift problem using a single SLOT (target label) sample generated with minimal manual effort. The selected SLOT samples effectively captured the core characteristics of the target domain, enabling the model to adapt efficiently to the target environment while significantly reducing the burden of manual labeling.
2. A DAS based on Genetic Algorithms (GAs) was applied to optimally augment the data derived from the SLOT sample, thereby constructing a more accurate base model. The automated exploration of the augmentation parameters improved the model’s performance in a stable and efficient manner.
3. A novel super-low-threshold strategy, previously unexplored in existing self-training approaches, was introduced to incorporate pseudo-labels with low confidence scores into the training process. Owing to the high accuracy of the base model achieved in the SLOT+DAS stage, this approach was able to suppress excessive false detection noise while further enhancing the domain adaptation performance.
4. Integrating these components into a unified system confirmed that the model consistently maintained high accuracy even under varying real-world deployment conditions (e.g., lighting, camera angles, and background). This integration is considered a key factor that increases the practical applicability of the system in operational settings such as livestock farms.

The remainder of this study is structured as follows: Section 2 summarizes the previous studies on domain shift in pig detection. Section 3 describes the proposed domain-adaptive pig detection system. Section 4 presents the experimental setup and analyzes the results. Section 5 presents ablation studies to examine the effectiveness of each component in the proposed system. Section 6 discusses the findings in depth, supporting the results. Section 7 highlights the limitations of the current study and suggests future research directions. Section 8 concludes the study.

## 2. Related Work

Object detection is one of the core areas of computer vision technology that mimics the human visual system to recognize and classify objects in surrounding environments [37]. With the advancement of deep learning technology, research on CNN-based object detection has been actively conducted and applied across various industries [38]. Deep learning-based object detection can be broadly categorized into the following two approaches: two-stage and one-stage detection.

Two-stage detectors first identify object locations using selective search and then classify objects within those locations through regression. Representative models include the R-CNN family, such as Fast R-CNN [39] and Faster R-CNN [20]. While these models achieve high accuracy, they do not guarantee fast execution speeds. In contrast, one-stage detectors predict object locations and classes in a single operation without a separate candidate region generation process. Examples include You Only Look Once (YOLO) [21] and Single-Shot MultiBox Detector (SSD) [40], which focus more on speed rather than accuracy. Among them, YOLO models have been widely adopted in commercial applications due to their balance between accuracy and speed, continuously improving from YOLOv1 to YOLOv11 [21,41,42,43,44,45,46,47,48,49,50].

However, domain shift remains a critical issue when applying these models in commercial environments. The domain shift problem arises from differences in the data distributions between the trained source domain and the actual target domain, and this is a significant challenge in industrial applications such as pig object detection. In real livestock environments, domain shifts can occur due to various factors, including lighting conditions, camera placement, variations in individual pigs’ body shapes and colors, and differences in farming environments. To address this, domain adaptation techniques are required to enable models trained on the source domain to effectively adapt to the target domain effectively. This study aims to develop a practical detection system that can flexibly adapt to various environmental changes, such as lighting, camera placement, and pig appearance, which commonly occur on real livestock farms. This practical need for adaptability serves as a primary motivation for our work. Notably, this direction is also in line with recent neural network research. For example, Tufail et al. [51] emphasized the growing importance of models capable of self-adjusting to changing environments, highlighting a transition from static to dynamic architecture.

Table 1 summarizes pig object detection studies published from 2019 to the present that either report domain shift results (i.e., test performance on unseen data) [52,53,54,55,56,57] or apply semi-supervised learning methods [58,59]. Early studies demonstrated the domain shift problem by testing models trained solely on source domain data with unseen target data in a supervised learning manner [52,53,55]. Consequently, research efforts have been directed toward improving the generalization performance of object detection models, including Faster R-CNN- and YOLO-based models.

Among them, Zhang et al. [54] achieved a 12% relative performance improvement in mAP (from 0.75 to 0.84), the highest among supervised methods—by training source domain models with target-like data generated via a style transfer technique. However, domain shift remained unresolved despite such improvements, and models trained solely on source data struggled to achieve significant performance gains [56,57].

More recently, semi-supervised learning approaches that utilize target-domain data have been proposed to improve domain adaptation effectiveness. For example, Ref. [58] introduced the noisy student [33] training method, achieving a 37% relative performance improvement in the F1-score (from 0.67 to 0.92) using only unlabeled target-domain data, the highest accuracy recorded for pig monitoring without labeled target-domain data. Meanwhile, Ref. [59] added just ten labeled target-domain images, leading to a 61% relative performance improvement in AP (from 0.57 to 0.92), marking the highest accuracy gain reported to date. These results highlight that even minimal labeled target-domain data can significantly impact model performance.

In this study, we propose a novel self-training-based domain adaptation method that overcomes existing approaches’ limitations, which either avoids using labeled target data entirely or requires a relatively large amount of labeled target data. Specifically, considering that pseudo-label quality heavily depends on the initial base model’s performance, we introduce a method that utilizes a single labeled target image. The proposed method ensures a reliable initial base model for self-training, enhances pseudo-label quality, and maximizes domain adaptability.

## 3. Proposed Method

**Figure 2:**
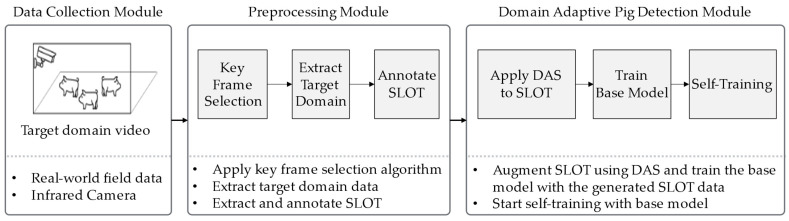
Diagram of the overall proposed structure on sustainable self-training pig detection system.

The proposed system consists of the following three modules: data collection, preprocessing, and domain-adaptive pig detection. Figure 2 shows the overall proposed system architecture.

### 3.1. Data Collection Module

In the Data Collection Module, data are gathered using camera sensors. Since this study collects data from an actual livestock farm operating, illumination conditions vary significantly between day and night. Low-light conditions may occur at night or on cloudy days. An infrared camera capable of capturing images in low-light environments was used to account for these variations. The camera was installed in a tilted view configuration to capture the vertically elongated rectangular pigpen. The collected target-domain data, which includes pigsty environments and pigs, serves as input for the Preprocessing Module. Detailed specifications of the camera and data acquisition process are provided in Section 4.1.

### 3.2. Preprocessing Module

Since pigs spend approximately 80% of their day resting [60], using raw data without refinement may lead to redundant images, reducing training efficiency. Therefore, the preprocessing module applies a key frame selection algorithm to minimize data redundancy and enhance the efficiency of the proposed method.

#### 3.2.1. Key Frame Selection

Key frame selection algorithms are generally used to efficiently compress entire videos by capturing meaningful changes and removing redundant frames. Gao et al. [61] proposed extracting key frames from surveillance videos by calculating entropy and applying the K–means algorithm to the results. Their experiments demonstrated that entropy calculation alone, without semantic information from the video (e.g., voice), effectively detect changes within the footage. In this study, we simplify the approach of [61] by extracting key frames using only entropy calculations. The simplification reduces the computational burden of the conventional method while allowing key frames to be selected quickly and intuitively.

To compute entropy, the image is first converted to grayscale, and the frequency of each pixel intensity value is calculated. Let me generate the following example implementation for this process:

(1) $$\begin{array}{c} p_{i}\left(l\right)=\sum_{y=1}^{H-1}\sum_{x=1}^{W-1}\left|f_{i}\left(y,x\right)=l\right| \end{array}$$

here, *H* and *W* represent the vertical and horizontal coordinates of the image, respectively, and $f_{i}(y,x)$ denotes the pixel value of the image in the *i*th-frame. Based on the result of Equation (1), the entropy of the corresponding frame is then calculated as follows:

(2) $$\begin{array}{c} e_{i}=-\sum_{l=0}^{255}p_{i}\left(l\right)\log p_{i}\left(l\right) \end{array}$$

here, K represents grayscale values ranging up to 255. As a result, each image can be represented by a single entropy value, and a video consisting of N frames can be expressed as a set as follows:

(3) $$\begin{array}{c} V_{ent}=\left[e_{0},e_{1},\ldots ,e_{N-1},e_{N}\right] \end{array}$$

The difference between entropy values is calculated to extract key frames. Specifically, if the difference between the previously stored entropy value and the entropy value of the current frame exceeds a predefined threshold $\tau_{ent}$, the frame is considered to contain a significant change and is stored. The extracted key frames are then used as unlabeled target-domain data.

#### 3.2.2. Single Label on Target Data Selection

Once the target-domain data are extracted through key frame selection, the next step is to select SLOT, which plays a crucial role in the proposed system. Here, SLOT refers to a single manually labeled image from the target domain, which serves as the minimal supervision signal for domain adaptation in our framework. The extracted key frames are sorted based on their entropy values to select SLOT. Then, the single image with the highest entropy value is chosen as the representative SLOT data. Selecting the frame with the highest entropy ensures inclusion of diverse content (e.g., pigs in varied postures and positions), making it a strong representative of the target domain with minimal annotation. Finally, a human annotates the selected SLOT data, which are then utilized in the domain-adaptive pig detection module.

### 3.3. Domain-Adaptive Pig Detection Module

This module aims to train a model that resolves the domain shift problem by utilizing the target-domain data extracted through the preprocessing module and the SLOT data. The model training process consists of two stages. In the first stage, the DAS method, which employs a GA-based search for optimal augmentation parameters, is applied to the SLOT data to train the base model. In the second stage, the trained base model is used as the initial model for applying self-training. Finally, an algorithm that integrates both training stages into a unified process is proposed.

#### 3.3.1. Data Augmentation Search for Base Model Training

The proposed system maximizes data augmentation techniques to train the base model from a single SLOT dataset effectively. However, testing all possible combinations is inefficient and practically impossible. Therefore, DAS is applied to search for the optimal data augmentation parameters automatically. DAS consists of the following three components: search space, algorithm, and estimation metric. The search space refers to the set of data augmentation parameters to be explored; the search algorithm is used to find the optimal parameters within the search space; and the estimation metric evaluates the performance of the explored parameters.

**Figure 3:**
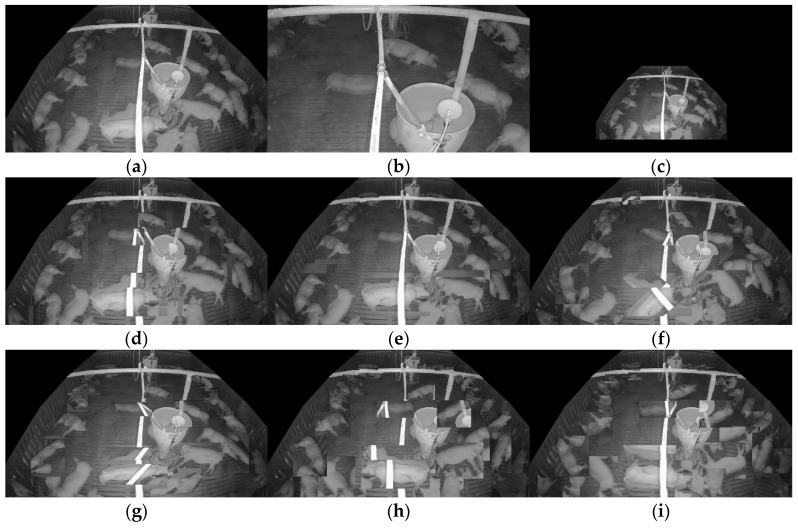
Examples of augmentation using GA-based augmentation: (**a**) original image; (**b**) zoom-in; (**c**) zoom-out; (**d**) translateX; (**e**) translateY; (**f**) result of rotation; (**g**) result of shearing; (**h**) flipH; (**i**) flipV.

First, the search space defines the augmentation parameters to be explored. The parameters to be searched in the proposed system are listed in Table 2. $N_{image}$ determines the diversity and size of the dataset. Zoom-in/-out enables the learning of objects at various scales and facilitates learning objects from different perspectives. Here, zoom-in/-out is applied to the entire image frame, whereas image transformation is applied to individual objects. Figure 3 illustrates applicable augmentations depending on the activated image transformation flag.

**Figure 4:**
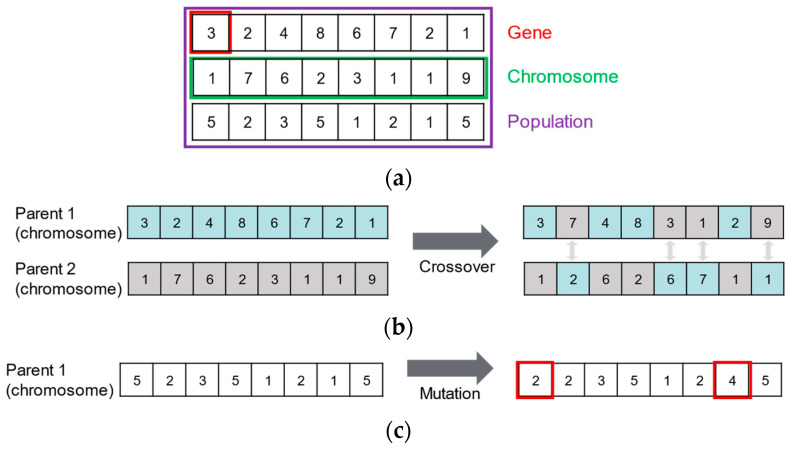
The GA’s components and operations: (**a**) visually shows the components of the GA, including the gene, chromosome, and population; (**b**) visually illustrates the crossover operation, one of the processes in the GA; (**c**) visually illustrates the mutation operation, another process in the GA.

Once the search space is defined, it is necessary to determine which search algorithm will be used for exploration. Various search algorithms exist, but this study employs a GA, which can provide multiple reasonable solutions rather than a single solution for discontinuous data. This is particularly important in our setting, where the augmentation policy consists of multiple discrete and interdependent parameters (e.g., zoom type, transformation flag, and magnitude). Compared to brute-force strategies, the GA is known to be more effective in exploring high-dimensional combinatorial spaces due to its population-based and stochastic search mechanisms [62]. The GA consists of genes, chromosomes, and a population. A gene represents each parameter to be applied, while a chromosome represents a set of parameters. These chromosomes collectively form a population. The core procedure of the GA is as follows: (1) Randomly initialize the initial population; (2) evaluate the fitness of the parent chromosomes; (3) select only the chromosomes with high fitness to serve as parents for the next generation; and (4) generate new offspring chromosomes through crossover and mutation operations based on the selected parent chromosomes. Here, crossover refers to an operation that exchanges portions of genes between two parent chromosomes to create a new offspring chromosome. At the same time, mutation is an operation that randomly modifies a chromosome’s genes, typically with a low probability. Repeat steps 2–4 to explore the optimal augmentation parameters. Figure 4 visually illustrates the GA’s key components and operational processes. Algorithm 1 shows the pseudo-code of the GA.
**Algorithm 1.** Pseudo-code of the GA**Input:** Objective function: f(x), Population size: P, Number of generations: G**Output:** Best solution: $c^{*}$
**Initialize:** Initialized population: $P_{0}\leftarrow \left\{c_{1},c_{2},\ldots ,\;c_{P}\right\}$
1for g←1 to G
2  for each $c_{k}\in P_{g-1}$
3    $f_{k}\leftarrow f\left(c_{k}\right)$ -- evaluate fitness4    $F\leftarrow F\cup \left\{\left(c_{k},f_{k}\right)\right\}$
5  $P_{elite}\leftarrow SelectTopP(F,P)$ -- select top P chromosomes with highest fitness6  $P_{cross}\;\leftarrow \;Crossover\left(P_{elite}\right)$
7  $P_{g}\leftarrow Mutation\left(P_{cross}\right)$
8$c^{*}=\underset{(c_{k},f_{k})\in F}{\mathrm{argmax}}f_{k}$

Finally, the estimation metric is used to evaluate the model’s accuracy achieved when trained with the augmented data and to explore the optimal parameters. In this study, AP, which has already been validated as effective in a previous study [63], is used as the estimation metric. However, since no labeled target-domain data are available, it was necessary to construct evaluation data independent of the augmentation candidates used in the DAS. To address this, we introduced the copy-and-paste technique [64,65] to generate independent evaluation images explicitly excluded from the DAS parameter search process. This approach ensures that the evaluation data do not overlap with the training data, preventing overfitting during augmentation parameter optimization. Furthermore, we randomly varied the position and number of pasted objects to minimize the risk of overfitting the specific objects derived when generating the evaluation dataset. This process introduces high variability in object context and density, thereby increasing the diversity of the evaluation set and making it more reflective of realistic and unseen scenarios. By ensuring that each synthesized image contained different object placements and counts, we reduced the likelihood of overfitting to specific object instances and enhanced the robustness of the fitness evaluation.

The copy-and-paste technique extracts objects from labeled images and pastes them onto background images to augment the data. Object extraction is possible using SLOT data in the proposed method, but background images are unavailable. Therefore, the proposed system generates background images by calculating each pixel’s cumulative moving average (CMA) from easily obtainable monitoring footage. Equation (4) represents the formula used for the background image generation.

(4) $$\begin{array}{c} f_{background}\left(y,x\right)=\frac{1}{total\;frame}\sum_{n=0}^{n=total\;frame}f_{n}\left(y,x\right) \end{array}$$

where *y* and *x* represent the pixel values corresponding to the vertical and horizontal positions, respectively, while $f_{n}$ denotes the nth frame. As a result, objects extracted from the SLOT data were copied onto the background images generated using Equation (4) to create new evaluation images.

**Figure 5:**
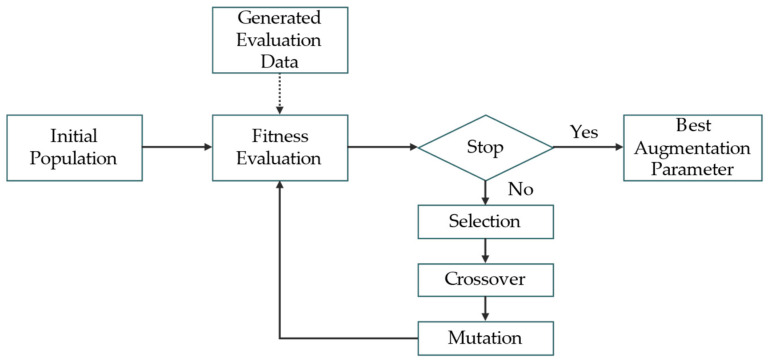
Diagram of the DAS method’s architecture.

As shown in Figure 5, the DAS explores the optimal SLOT data augmentation parameters within the predefined search space using a GA. In this process, the fitness of each chromosome is evaluated based on the AP of a detector trained for 30 epochs using a dataset composed of source images, SLOT images, and SLOT images augmented based on the given chromosome. An independent validation dataset generated using the copy-and-paste technique was employed for the fitness evaluation. Early stopping [66] was applied during model training to ensure efficient AP calculation to reduce the training time. Early stopping terminates training if the validation loss does not decrease for E consecutive epochs, preventing overfitting and enabling efficient learning. Finally, the SLOT data were augmented based on the parameters that achieved the highest fitness among the explored parameters. Consequently, the base model was trained using source, SLOT, and augmented SLOT data. The trained base model was then used as the initial model for self-training.

#### 3.3.2. Self-Training to Address Domain Shift

Self-training is a method that progressively improves a model by utilizing the pseudo-labeling technique, allowing it to adapt even to a target domain without labeled data [67]. Self-training was adopted in this study due to its architectural simplicity, high compatibility with low-label environments, and ease of integration into existing object detection frameworks. Unlike adversarial domain adaptation methods that require complex loss functions and auxiliary components [68], self-training improves model performance through iterative learning solely using target-domain data, making it more reproducible under real-world constraints such as limited labeling in commercial pig farms. Moreover, because self-training is a training strategy rather than an architectural module, it can be applied directly to state-of-the-art object detectors without modifying the backbone or detection head. This architectural independence ensures broad compatibility and supports rapid deployment across various detection models.

**Figure 6:**
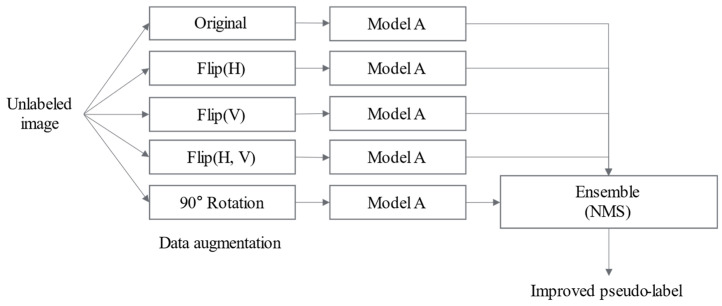
A diagram illustrating the image transformation techniques used in DD. The image transformations include original, horizontal flip, vertical flip, horizontal and vertical flip, and 90° rotation.

In this study, self-training is performed using the previously trained base model as the initial model, ensuring that the model learns the features of the target data. The key aspect of self-training is increasing the diversity and reliability of pseudo-labels. The proposed system applies data distillation (DD) [69], which enhances diversity through scaling and horizontal flipping. Vertical flipping and 90-degree rotation transformations are incorporated into the self-training process. Figure 6 illustrates the DD method applied in the proposed system.

The proposed system’s DD technique follows these steps. First, the base model detects unlabeled target data extracted by the preprocessing module. During detection, DD is applied to generate various transformed image results, which are then ensembled to assign bounding boxes. Then, among the predicted bounding boxes, those with confidence scores above the predefined reliability threshold are assigned as pseudo-labels. Next, the final target model is trained using SLOT, source, and pseudo-labeled target-domain data. Finally, these steps are repeated to train a more accurate model progressively.

In this process, the method for pseudo-label filtering plays a critical role. A confidence-based filtering scheme is employed to determine which predictions are used as pseudo-labels. That is, bounding boxes with scores above a predefined threshold are selected and used in the training. Specifically, each target image is first transformed through multiple geometric augmentations, such as horizontal and vertical flipping and rotation, as part of the DD process. Object detection is performed on each transformed version, and the predicted bounding boxes are mapped back to the original coordinate space. These predictions are then aggregated using non-maximum suppression (NMS), which identifies overlapping boxes across different transformed views and retains only the one with the highest confidence score. The retained bounding boxes are then compared against the predefined confidence threshold, and those exceeding the threshold are selected as pseudo-labels for self-training.

Most conventional self-training approaches select pseudo-labels based on high confidence thresholds to minimize label noise [70,71,72,73,74]. In contrast, this study adopts a super-low-threshold strategy. This choice is grounded in the hypothesis that, following the DAS phase, the performance of the initial base model is sufficiently improved, making even low-confidence predictions potentially beneficial for learning.

Accordingly, we significantly lowered the confidence threshold to maximize the number of pseudo-labels, thereby encouraging the model to learn from a broader distribution of domain-specific information. While this approach may introduce some noise, we argue that the expected gains in generalization, achieved through exposure to a more diverse range of samples, outweigh the potential drawbacks.

In summary, the proposed system was designed to address domain shift while minimizing manual labeling. Initially, SLOT provided essential domain-specific information with minimal effort. Then, the data diversity was expanded through genetic algorithm-based DAS to strengthen the base model. Finally, a super-low-threshold strategy was adopted in the self-training to maximize the domain-adaptation capability by learning from a broader range of target-domain pseudo-labels. This progressive design enables effective domain adaptation with minimal human supervision. As a result, the domain-adaptive pig detection module follows the algorithm outlined in Algorithm 2.
**Algorithm 2.** SLOT-DAS with Self-Training for Domain Adaptation**Input:** Source data: $D_{source}\leftarrow \left\{\left(x_{1},\;y_{1}\right),\;\left(x_{2},\;y_{2}\right),\ldots \;,\;\left(x_{N},\;y_{N}\right)\right\}$, SLOT data: $D_{SLOT}\leftarrow \left\{\left(x_{SLOT},\;y_{SLOT}\right)\right\}$, Copy-and-paste data: $D_{CP}\leftarrow \left\{\left(x_{1},\;y_{1}\right),\;\left(x_{2},\;y_{2}\right),\ldots ,\;\left(x_{L},\;y_{L}\right)\right\}$, Unlabeled target-domain data: $D_{target}\leftarrow \left\{x_{1},\;x_{2},\;...\;,\;x_{M}\right\}$**Output:** Best augmentation parameter: $c^{*}$, Trained target model: $M^{*}$
**Initialize:** Number of generations: G, Initialized population: $P_{0}\leftarrow \left\{c_{1},c_{2},\ldots ,\;c_{K}\right\}$, Set of chromosomes: C←∅, Self-training iteration: I, Confidence threshold: $\tau_{conf}$, Set of DD transformations: $T\leftarrow \left\{t_{1},\;t_{2},\;...,\;t_{Q}\right\}$
1for g←1 to G
2  for each $c_{k}\in P_{g-1}$
3    $A_{k}\leftarrow Augment\left(D_{SLOT},\;c_{k}\right)$
4    $D_{k}\leftarrow D_{source}\cup D_{SLOT}\cup A_{k}$
5    $M_{k}^{*}\leftarrow Train\left(M,D_{k}\right)$
 -- early stop applied6    $f\left(c_{k}\right)\leftarrow Evaluate\left(M_{k}^{*},D_{CP}\right)$
 -- using AP7    $C\;\leftarrow C\cup \left\{\left(c_{k},f\left(c_{k}\right)\right)\right\}$
8  $P_{elite}\leftarrow SelectTopK(C,K)$
9  $P_{cross}\;\leftarrow \;Crossover\left(P_{elite}\right)$
10  $P_{g}\leftarrow Mutation\left(P_{cross}\right)$
11$c^{*}=\arg\underset{c_{k}}{\max}f\left(c_{k}\right)\;$12$D_{A^{*}}=Augment\left(D_{SLOT},c^{*}\right)$13$D_{base}=D_{source}\cup D_{SLOT}\cup D_{A^{*}}$14$M_{0}=Train\left(M,D_{base}\right)$15$M_{s}\leftarrow M_{0}$16for i←1 to I
17  $D_{pseudo}\leftarrow \emptyset$
18  for each $x_{m}\in D_{target}$
19    $x_{m}^{t}=t\left(x_{m}\right),\;\forall t\in T$
20    ${\hat{y}}_{m}^{t}=M_{s}\left(x_{m}^{t}\right),\;\forall t\in T$
21    ${\hat{y}}_{m}=Ensemble\left({\left\{{\hat{y}}_{m}^{t}\right\}}_{t\in T}\right)$
22    ${\hat{y}}_{m}^{pseudo}=\left\{y\in {\hat{y}}_{m}|{score}_{conf}\left(y\right)\geq \tau_{conf}\right\}$
23    $D_{pseudo}\leftarrow D_{pseudo}\cup \left\{\left(x_{m},\;{\hat{y}}_{m}^{pseudo}\right)\right\}$
24          $D_{train}^{i}\leftarrow D_{source}\cup D_{SLOT}\cup D_{pseudo}$
25  $M_{s}=Train\left(M_{s},D_{train}^{i}\right)$
26$M^{*}=M_{s}$

## 4. Experiments

### 4.1. Data Collection and Datasets

**Figure 7:**
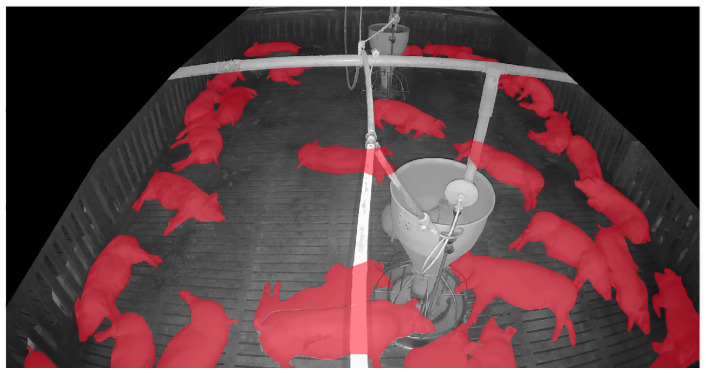
Annotation result for the SLOT data after the key frame selection algorithm’s application.

The proposed system trains and evaluates the model using data collected from actual commercial pig farms. The base model training utilizes source domain data and SLOT data, while the self-training process additionally incorporates unlabeled target-domain data. Model evaluation is conducted using independent data collected from the target domain. The frame with the highest entropy value was selected as SLOT data. Table 3 summarizes the data used for training and testing, while Figure 7 presents the annotated SLOT data.

The source domain data consist of data from commercial pig farms in Hamyang and Jochiwon, the Chungbuk data collected from Chungbuk University, and an open dataset from AI Hub [75]. These datasets were captured using top-view cameras installed to observe pig behavior. A total of 5961 frames from the entire source domain dataset were used.

The target-domain data were collected from a commercial pig farm in Hadong, Gyeongsangnam-do, South Korea. These data were recorded using an infrared dome camera (QND-6012R, Hanwha Techwin, Changwon, Korea) installed on the ceiling of the pigsty from 26 October to 27 October 2021, for approximately 25 h. The video was recorded at 10 frames per second (fps) with a resolution of 1920 × 1080. The camera was installed with a tilted view to capture the rectangular pigsty comprehensively. The collected data reflect commercial farm conditions, including day and night transitions and pig movements. The target-domain data underwent preprocessing to be extracted as unlabeled target-domain data and SLOT data.

During preprocessing, the pre-threshold, $\tau_{ent}$, for the key frame extraction algorithm was set to 0.05, resulting in 4000 key frames being extracted from 90,000 frames. All 4000 extracted key frames from the target domain were manually annotated for evaluation to establish a fully supervised benchmark (Oracle). However, during the domain adaptation training phase, only one of these annotated images was used as the labeled target (SLOT). In contrast, the remaining 3999 images were treated as unlabeled data for self-training. This setup ensures a fair comparison across methods; all approaches were evaluated on the same annotated target dataset, but our method uses only one labeled frame for training. This benchmark protocol allows us to isolate the effectiveness of our approach.

### 4.2. Experimental Environment and Setup

#### 4.2.1. Implementation Details

The proposed system was implemented with a single GPU (GeForce RTX 2070, NVIDIA Corporation, Santa Clara, CA, USA), and for each model, the batch size was adjusted to the maximum value that could be trained without exceeding the GPU’s memory capacity. YOLOv8-m was utilized as the object detection model to verify the effectiveness of the proposed system. Although new YOLO-based models continue to emerge, the latest models do not necessarily guarantee a better performance, as their effectiveness can vary depending on specific use cases [76]. Therefore, this study selected YOLOv8, which has been widely validated and adopted [77,78,79]. Among the YOLOv8 model structures (s, n, m, l, x), YOLOv8-m was chosen as it is known to provide the best balance between accuracy and training time [78,79], making it the most suitable option for small- to medium-sized farms by ensuring high accuracy while optimizing computational resources. Nonetheless, exploring the applicability of newer architectures, such as YOLOv12 [80], may be a valuable direction for future research to assess potential improvements in performance or efficiency under different domain conditions.

For comparative experiments, models with similar parameter sizes, including YOLOv5-m, YOLOv6-s, and YOLOv7, were used to analyze performance differences. The input image size for all models was fixed at 640, and all other hyperparameters were kept at their default values to ensure a fair comparison [44,45,46,47].

In the DAS, the search space was constrained, as shown in Table 4, to enable efficient exploration. The parameter settings used in this study, including the probability and magnitude ranges for zoom-in and zoom-out, were carefully determined concerning prior work [63]. In particular, to ensure that the original image content is preserved to a certain extent, the upper bound of the probabilities for both zoom-in and zoom-out was limited to 0.5. Other parameters were configured based on experimentally validated ranges reported in a previous study, considering visual plausibility and computational efficiency. $N_{image}$ was set to be selected as an integer between 10 and 500. The probability $P_{zoom\_in/out}$ for zooming in and out was set to be within the range of 0 to 0.5, while the probability of using the original image was calculated as $P_{original}=1-P_{zoom\_in}-P_{zoom\_out}$. The magnitude, $M_{zoom\_in}$, in zoom-in/-out was searched within the range of 1.2 to 1.9, and $M_{zoom\_out}$ was searched within the range of 0.2 to 0.9. $F_{trans}$ was selected as one of six flags (0–6), and the probability, $P_{trans}$, was set between 0.1 and 1. The magnitude, $M_{trans}$, was assigned values between 1 and 8 depending on the selected flag. If the selected flag was 0, $P_{trans}$ and $M_{trans}$ did not affect the results and were fixed at 1. Additionally, if the flag was set to 5 (flipH) or 6 (flipV), $M_{trans}$ was undefined and was, therefore, fixed at 1. Consequently, the total search space to be explored was approximately $491\times 6^{2}\times 8^{2}\times \left(10\times \left(4\times 8+2\right)+1\right)\approx 3.85\times 10^{8}$.

The parameters used in the proposed algorithm are as follows: The number of generations, G, for the DAS was set to 3, and the population size, K, was set to 20. In each generation, crossover and mutation were performed 10 times each. These parameters were set based on practical considerations aimed at maintaining a sufficient level of diversity within the search space while avoiding excessive computational costs. Although systematic hyperparameter tuning was not conducted, the configuration was deemed appropriate given the constraints and requirements of the intended application environment. The model trained for fitness evaluation was trained for up to 30 epochs, with the early stopping epoch, E, set to 3. The number of iterations, I, for self-training was set to 5, with each iteration trained for 5 epochs. The confidence threshold, $\tau_{conf},$ for pseudo-label generation was set to 0.01. As a result, the base model was trained for 150 epochs, while self-training continued for up to 175 epochs.

#### 4.2.2. Evaluation Metric

By standard practices in the computer vision field, we adopted AP as the primary evaluation metric for our object detection task. AP jointly reflects precision and recall, thereby offering a comprehensive measure of detection performance by accounting for both false positives and false negatives. In addition to its widespread use in general object detection tasks, AP is commonly employed in domain shift studies to compare detection accuracies between source and target domains [53,54,55,57,59]. Furthermore, since the self-training framework adopted in this study is susceptible to the quality of pseudo-labels, using AP based on prediction confidence enables fine-grained monitoring of model performance and pseudo-label reliability throughout the training process. In our study, AP serves two roles. It is used as the fitness function during the DAS process and as the main evaluation metric for assessing the accuracy of the object detection model.

The computation method for AP is presented in Equation (5). It is obtained by dividing the recall values from 0 to 1 into 11 points and computing the average of the precision values corresponding to these recall values. In Equation (6), $p\left(\tilde{r}\right)$ represents the precision value corresponding to a specific recall value on the precision–recall curve. The precision at each point is defined as the highest precision value among recall values greater than or equal to the given recall value. Precision and recall are defined in Equations (7) and (8), respectively. Here, true positive (TP) refers to the number of predicted boxes that correctly detect objects, false positive (FP) represents the number of predicted boxes that fail to detect objects, and false negative (FN) denotes the number of objects that were not detected.

(5) $$\begin{array}{c} AP=\frac{1}{11}\sum_{r\in \{0,0.1,\ldots ,1\}}p_{interp}\left(r\right) \end{array}$$

(6) $$\begin{array}{c} p_{interp}\left(r\right)=\underset{\tilde{r}:\tilde{r}\geq r}{\max}p\left(\tilde{r}\right) \end{array}$$

(7) $$\begin{array}{c} Precision=\frac{TP}{TP+FP} \end{array}$$

(8) $$\begin{array}{c} recall=\frac{TP}{TP+FN}\; \end{array}$$

### 4.3. Domain-Adaptive Pig Detection Results

#### 4.3.1. Data Augmentation Search Results

The proposed system utilized the copy-and-paste technique to generate evaluation data required for the DAS process. In this process, background images were created using the cumulative moving average of input frames, while objects were extracted from SLOT data.

**Figure 8:**
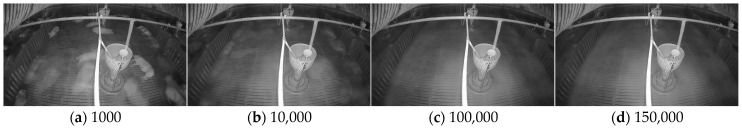
Results of the background images generated by calculating the cumulative moving average of each pixel: (**a**) 1000 images; (**b**) 10,000 images; (**c**) 100,000 images; (**d**) 150,000 images.

Figure 8 illustrates the progressive changes in the background images extracted. Figure 8a presents the result obtained using 1000 images, where some objects remain distinguishable. Figure 8b shows the result with 10,000 images, where object traces have diminished, although static objects retain their shapes. Figure 8c depicts the result using 100,000 images, showing a further reduction in object traces and a more transparent background. Finally, Figure 8d demonstrates the outcome with 150,000 images, where nearly all moving objects have entirely disappeared from the background, resulting in a highly stable background image. Consequently, Figure 8d was selected as the background for the copy-and-paste technique.

**Figure 9:**
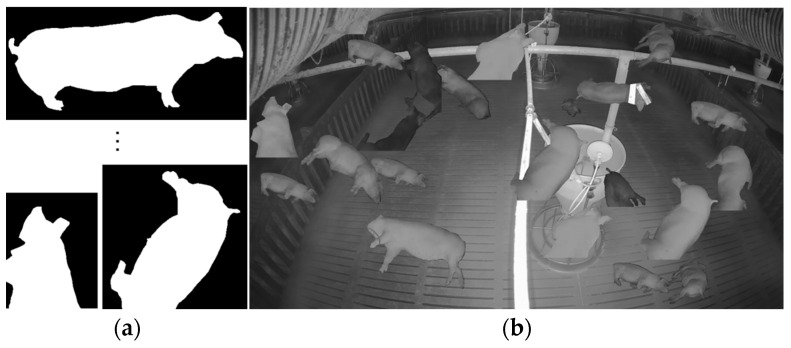
SLOT individual object annotation information and copy-and-paste application example: (**a**) annotation information of objects extracted from SLOT data; (**b**) evaluation data generated using the copy-and-paste technique.

Figure 9 presents examples of SLOT annotations and the application of the copy-and-paste technique. Figure 9a displays annotation details of individual objects extracted from SLOT data, while Figure 9b shows an example of evaluation data generated by copying SLOT objects onto the background image. Ultimately, 100 images generated through the copy-and-paste technique were used as evaluation data for the DAS.

Based on the previously generated evaluation data, DAS was applied to explore the optimal augmentation parameters within the search space. Table 5 presents the results of applying DAS to SLOT data using YOLOv8. This table displays the top two parameter settings with the highest AP scores (TOP1 and TOP2) and the bottom two with the lowest AP scores (TOP59 and TOP60).

For $N_{image}$, the values for TOP1 and TOP2 were 48 and 140, respectively, which are significantly lower than the values of 337 and 388 for TOP59 and TOP60. This indicates that an excessive number of DAS-generated images can lead to unnecessary complexity in the dataset. If too many images are created, the model may learn redundant information or noise from diverse data, which can degrade performance.

For the $P_{zoom\_in}$ parameter, TOP1 was set to 0, meaning zoom-in was not applied, while TOP2 was set to 5, meaning it was frequently activated. This suggests that the impact of zoom-in may have been offset by the influence of other augmentation parameters, which acted complementarily. As a result, zoom-in appeared to have a weaker effect on performance compared to other parameters. On the other hand, for $P_{zoom\_out}$, TOP1 and TOP2 exhibited high values and achieved high AP scores. This indicates that zoom-out positively contributed to model training, and accuracy improvements could be expected when smaller objects were trained with higher frequency.

Regarding the $F_{trans}$ parameter, TOP1 was set to 0 (original), while TOP59 and TOP60 were set to 6 (flipV) and 3 (rotate), respectively. This suggests that excessive transformations may have distorted the visual characteristics of objects, leading to performance degradation.

The experimental results not only identified the most suitable augmentation parameters for the SLOT data but also provided insights into how each augmentation affected the accuracy through analysis of the search results.

#### 4.3.2. Self-Training Results

Table 6 presents the results of training the base model used as the initial model for self-training, incorporating various training methods and YOLO series models. The Oracle method represents supervised learning results using all 4000 labeled target-domain data. The source-only method represents the model trained without using target data. The SLOT method represents the training results using source data with the addition of only one SLOT data instance. The DAS method extends the SLOT method by incorporating augmented SLOT data generated by DAS.

In the Oracle results, YOLOv7 and YOLOv8 achieved the highest accuracy. However, YOLOv6’s accuracy was lower than that of the DAS method. This accuracy could be attributed to overfitting during the 150-epoch training process.

The source-only results show the lowest accuracy across all models. YOLOv8 recorded the lowest accuracy at 36.86, significantly lower than the other models. This result suggests that applying the model directly to real-world commercial settings without adaptation to the target domain would likely result in high numbers of false positives and false negatives. In the SLOT results, YOLOv8 exhibited the highest improvement, achieving a performance increase of 43.39 compared to the Source-only method. The DAS results demonstrate performance improvements across all models without requiring additional human labor, with a maximum accuracy gain of 7.68.

Table 7 summarizes the results of self-training and DD for each model based on the threshold values and epochs. The 150-epoch model corresponds to the base model trained using DAS, as shown in Table 6. The experimental results show that the accuracy also improved as the numbers of iterations in the self-training and DD increased. The highest accuracy was observed when applying the super-low threshold ($\tau_{conf}=0.01$). This trend was particularly prominent in YOLOv8. For instance, when using a threshold of 0.5 to generate pseudo-labels, applying the super-low threshold resulted in accuracy improvements of 4.65 in self-training and 4.15 in DD. Additional self-training results across the YOLOv5, YOLOv6, and YOLOv7 models are summarized in Appendix A, further supporting the generalizability of the proposed framework.

Table 8 summarizes the overall experimental results based on the YOLOv8 model. The most significant performance improvement was observed when SLOT data were added. Additionally, incorporating augmented data generated through DAS resulted in an approximately 5% increase in performance. In the DD results, using a threshold of 0.5 to generate pseudo-label data led to only a 1.20 improvement in performance. However, when applying the super-low threshold, a performance gain of 5.35 was achieved compared to the DAS results.

Table 9 compares the results of different self-training techniques across models, including the proposed system, naive self-training, and DD for YOLOv6, YOLOv7, and YOLOv8. The experimental results show that with YOLOv7 and YOLOv8, applying DD achieved higher accuracy than naive self-training. In contrast, the DD model performed worse than naive self-training with YOLOv6. The proposed system achieved the highest accuracy across all models, with the most significant performance improvement observed in YOLOv8 compared to DD.

Model training was initialized from a source-only baseline without using target-labeled data, including SLOT, for both naive self-training and DD. In the naive self-training, pseudo-labels were generated using a fixed confidence threshold of 0.5. For DD, we implemented a simplified version of the original method by applying multi-view augmentation and ensemble prediction, while replacing the per-category thresholding strategy employed in the original DD approach with a fixed threshold of 0.5 for pseudo-label selection. This unified threshold setting allows for a fair comparison between the two self-training approaches under consistent pseudo-label filtering criteria.

Table 10 presents the experimental results verifying whether the proposed system effectively operates under various domain shift conditions. This experiment was conducted in the reverse scenario of the previous top-view → tilted-view experiment to examine whether the proposed method can also effectively address domain shift when transitioning from a tilted view to a top view. For the experiment, Hadong data, corresponding to the tilted view, were used as the training dataset, while AI Hub data, corresponding to the top view, were used as the test dataset. A sample of 1000 images was randomly selected from the test dataset to maintain consistency in the evaluation environment.

The experimental results show that the Source-Only model exhibited a decline in performance due to domain shifts. The greatest performance improvement was observed when applying the SLOT method. The proposed system achieved a performance gain of approximately 20 compared to the Source-Only model. Although this improvement was lower than the 53-point increase observed in the top-view → tilted-view experiment, it still demonstrates a significant enhancement.

Ultimately, the proposed method maintained a robust performance across various domain shifts, achieving an accuracy of 90.16. The accuracy experimentally confirms that the proposed system is not limited to specific domain shifts but can maintain stable performance under diverse environmental variations.

## 5. Ablation Studies

### 5.1. Effect of Entropy-Based SLOT Data Selection

The performance of the proposed system is significantly influenced by the base model, which is a key factor in determining the final model’s accuracy. Therefore, selecting the appropriate SLOT data for base model training is crucial. In this study, entropy values were used as the selection criterion to compare and analyze images from the target domain with varying entropy levels. Table 11 presents the base model training results based on SLOT data selected according to entropy values.

The results indicate that using images with higher-entropy values led to a better base model performance. These results can be attributed to high-entropy images containing more diverse features, providing more meaningful information during model training. In contrast, low-entropy images often contained more static backgrounds or lacked diversity, limiting their effectiveness in training the model. These findings suggest that selecting SLOT data with high-entropy values positively impacts the model’s performance. Ultimately, this improves the accuracy of the self-training and domain adaptation while ensuring a robust performance even in changing field environments.

### 5.2. Initial Model Performance Based on the Number of SLOT Data Samples

Experiments were conducted using varying amounts of SLOT data to investigate the impact of the number of SLOT data samples on model performance. YOLOv8 was used as the evaluation model, and only source and SLOT data were included in the analysis. The number of SLOT samples ranged from 0 to 4000, and the results are summarized in Table 12.

The results show that adding just a single SLOT sample significantly improved model performance, with AP increasing from 36.86 to 85.81. This demonstrates that even minimal labeled information from the target domain can lead to substantial performance improvements. While further increases in the number of SLOT samples continued to enhance performance, the improvement gradually diminished. For example, using five SLOT samples increased AP to 88.62, while using ten samples further improved it to 92.48. However, beyond this point, the rate of improvement slowed. Specifically, with 100 SLOT samples, AP reached 93.67, while using 1000 and 4000 SLOT samples resulted in AP scores of 95.26 and 95.78, respectively, indicating that performance gains approached saturation.

From a trade-off perspective, annotating bounding boxes for a single image, typically containing 15–25 objects, takes about 2–3 min of manual effort [29] and improves AP from 36.86 to 85.81, achieving a gain of approximately 49 points. However, further performance improvements require tens to hundreds of hours of additional manual labeling. For example, labeling 1000 images yields only an additional 10 percentage point gain. In contrast, DAS requires approximately 86 h of computation time and is a fully automated process with no human intervention. This approach replaces labor-intensive labeling with an automated computational procedure, providing a repeatable and scalable structure even as the data volume increases, demonstrating high efficiency and scalability.

These findings highlight that even a single SLOT sample can lead to a substantial improvement in model performance. The sharp increase in AP from 36.86 to 85.81 indicates that the model can effectively adapt to the target environment with minimal labeled information. This demonstrates that the proposed approach offers a cost-effective and efficient solution in real-world scenarios where obtaining labeled data is challenging.

### 5.3. Early Stopping in Data Augmentation Search

Table 13 compares the results of applying early stopping during DAS execution. In both cases, the number of generations, G, and chromosome, C, were set to 3 and 60, respectively, but differences were observed in the number of training epochs and total training time. Without early stopping, training continued for a total of 1800 epochs (60 × 30) epoch and took approximately 103 h. In contrast, with early stopping applied, training was completed in 1595 epochs, reducing the total training time to approximately 86 h. This resulted in a 16.5% reduction in the overall training time. Furthermore, the model trained with DAS-generated data achieved a higher AP (85.27) when early stopping was applied. This suggests that early stopping effectively prevented overfitting by terminating training at an optimal point, leading to better parameter selection.

As the DAS must perform iterative training over various augmentation combinations, it inherently requires relatively high computational resources. However, this process is executed only once during the initial model preparation stage. Once the optimal augmentation parameters are identified through this search, the resulting augmented SLOT dataset can be reused in subsequent self-training iterations or system redeployment without repeated searches.

In this study, applying the DAS led to a performance improvement with YOLOv8, raising the AP from 80.25 to 85.27—a gain of 5.02 points. Similar improvements were consistently observed across other object detection models, including YOLOv5, YOLOv6, and YOLOv7. These improvements demonstrate that the proposed DAS is not limited to a specific architecture but induces performance enhancement across all tested models, proving its high generalizability and reproducibility. Considering these performance improvements, the computational overhead incurred during the initial model-building phase is deemed acceptable and justifiable, even in practical application environments. Furthermore, by pre-limiting the search space, population size, and number of generations, the proposed framework maximizes the search efficiency relative to computational resources, achieving a practical balance between computational cost and long-term system stability. In cases where environmental changes are very frequent or repeated applications to new domains are required, the cumulative computational burden from repeated executions of the DAS should be carefully considered.

### 5.4. Effect of Key Framesz in Self-Training

Table 14 presents the self-training results comparing the application of the key frame selection algorithm. Specifically, it compares cases where pseudo-label data were generated using 4000 frames extracted at uniform intervals versus 4000 frames selected through the key frame selection algorithm. The comparison shows that applying key frame selection resulted in a performance improvement of 0.33 (90.29 → 90.62) regarding the highest accuracy achieved. The improvement indicates that, in addition to the threshold used for selecting pseudo-labels, the quality of the input images also plays a crucial role in model performance.

### 5.5. Performance Analysis of Image Transformation Techniques

Recent studies have demonstrated that applying image transformation techniques, such as perspective transformation [81], can mitigate the domain shift problem by reducing differences among image domains [81,82]. In this experiment, we compare and analyze the impact of the recently proposed perspective transformation technique [81] and the data generalization method (DOG) [82] on the accuracy of the proposed system. The perspective transformation in [81] enlarges distant objects that appear smaller in a tilted view, transforming them as if viewed from a top-down perspective. Meanwhile, DOG enhances the adaptability of object detection models through image generalization.

As shown in Table 15, when the proposed method was applied alone, the AP reached 90.62, demonstrating its effectiveness in mitigating the domain shift problem. When the perspective transformation technique was additionally applied, the AP improved to 90.73, indicating that image generalization through perspective transformation positively influenced performance. In contrast, when the DOG method was additionally applied, the AP decreased to 79.82. The method suggests that while the DOG method enhances model adaptability through data generalization, it may introduce noise in specific datasets, leading to performance degradation.

These results reaffirm that the perspective transformation technique contributes to alleviating domain shift while confirming that the proposed method maintains high detection performance even when combined with other image processing techniques. However, to effectively utilize the DOG method in future research, it will be crucial to establish a dedicated training strategy and optimize parameters through fine tuning.

## 6. Discussion

### 6.1. Correlation Between Entropy and the Number of Objects

**Figure 10:**
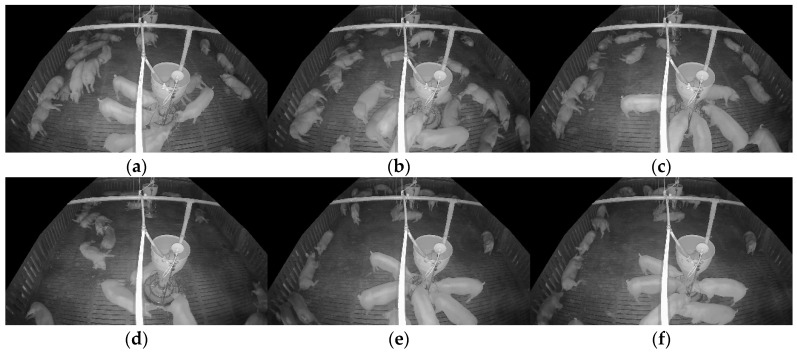
Examples of changes in the pigsty environment based on entropy values: (**a**) 0.8227; (**b**) 0.8225; (**c**) 0.8221; (**d**) 0.7975; (**e**) 0.7978; (**f**) 0.7985.

Figure 10 visually presents changes in the pigsty environment based on differences in entropy values. The experimental results show that images with high entropy contain a more significant number of pigs, providing more diverse visual information. In contrast, images with low entropy tend to include fewer pigs, reducing the complexity of the information. The dataset was divided into upper- and lower-entropy groups to quantitatively analyze this trend, and the average number of pig objects in each group was compared (Table 16). The analysis revealed that the upper-entropy group had an average of 26.5 pigs, whereas the lower-entropy group had an average of 19.7 pigs. The averages indicate that higher-entropy data generally include more objects, increasing the likelihood of learning diverse pig poses, sizes, and spatial arrangements during model training. Selecting high-entropy data can, therefore, contribute to more effectively reflecting the complexity of real-world farm environments, potentially leading to improved domain adaptation performance.

### 6.2. Data Augmentation Search Evaluation Data Generation Method

The evaluation data used in DAS were generated using the copy-and-paste technique, which can be categorized into object-based and segmentation-based methods. Determining which method is more suitable is essential. The object-based approach involves copying annotated data using bounding boxes onto the background image, while the segmentation-based approach uses polygon-annotated data for pasting.

**Figure 11:**
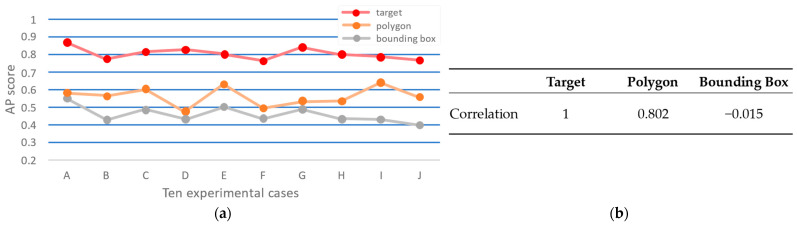
The correlation coefficient calculation results: (**a**) AP evaluation results of ten models using target data, polygon data, and bounding-box data. Each model on the x-axis was trained using a different augmented dataset generated with parameters selected by the DAS. The x-axis represents the individual models (labeled A–J), and the y-axis indicates the AP score; (**b**) correlation coefficient calculation results, from left to right, for target ↔ target, target ↔ polygon, and target ↔ bounding box.

The correlation coefficient between evaluation data generated using bounding boxes and polygons was measured against the target data to assess which method is more appropriate. Figure 11 presents the graph comparing the correlation coefficients. In Figure 11a, the x-axis represents ten experimental cases, each corresponding to a model trained using different augmented datasets generated with a distinct DAS-selected parameter. The y-axis indicates the AP score for each model. The results show that the polygon-based method achieved a higher correlation (0.802) with the target data, whereas the bounding-box-based method had a much lower correlation (−0.015). Thus, the final evaluation of the DAS results using the polygon-based method is more appropriate, as it more accurately reflects the characteristics of the target domain.

### 6.3. Comparison of Detection Results

**Figure 12:**
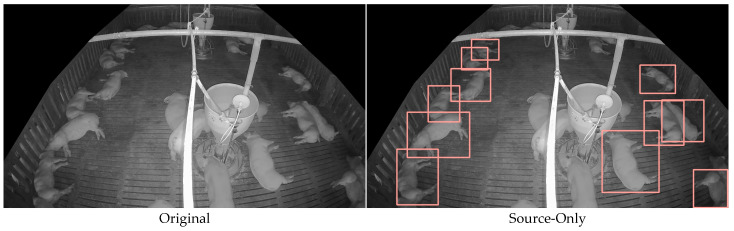

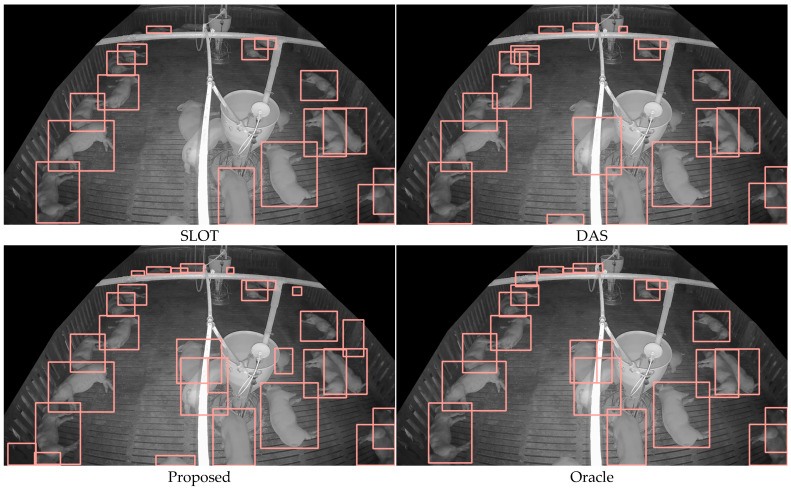
The object detection results for each model (Source-Only, SLOT, DAS, Proposed, and Oracle) are visually shown for the same image. The pink bounding boxes represent the objects detected by the models.

Figure 12 visually compares the object detection results of the trained models (Source-Only, SLOT, DAS, Proposed, and Oracle). The image contains 24 pigs, with a tilted view, causing objects at the top to appear smaller and those at the bottom to appear larger. The pink bounding boxes represent the objects detected by each model. The Source-Only model failed to detect standing pigs and smaller pigs compared to the other models, highlighting the training limitations with only source data. As the models progressed from SLOT → DAS → Proposed → Oracle, an improvement in accuracy was visually evident.

The Proposed model detected more objects than the Oracle model, demonstrating its ability to leverage a broader set of pseudo-labels through the super-low-threshold strategy. While this approach led to significant improvements in accuracy and adaptability under domain shift conditions, it also resulted in a higher number of false positives than other models, indicating a potential risk associated with noisy pseudo-labels. In particular, incorporating low-confidence predictions into training may increase the likelihood of including uncertain or inaccurate detections, which can introduce label noise and reduce precision in complex environments. Although the system maintained high AP across all experimental scenarios, this mechanism inherently carries a level of uncertainty that may affect performance when deployed in more diverse or dynamic real-world settings. Therefore, further refinement of the pseudo-label selection process may help mitigate residual false positives and enhance the robustness and consistency of the model across a broader range of deployment environments.

### 6.4. Sensitivity and Robustness Analysis of the Confidence Threshold

In the self-training process, the confidence threshold serves as a key hyperparameter that determines the criteria for pseudo-label collection, thereby influencing both the quantity and quality of the labels. Accordingly, analyzing performance variations under different threshold conditions is a critical step in validating the design soundness and robustness of the proposed approach.

In this study, we employed seven threshold values (0.001, 0.005, 0.01, 0.05, 0.1, 0.3, and 0.5) and summarized the resulting AP trends over self-training iterations (epochs 155–200) in Table 17.

The analysis revealed that most threshold configurations reached their maximum AP before epoch 175, with performance deterioration observed when training continued beyond a certain point. Specifically, thresholds of 0.001 and 0.005 achieved peak accuracies of 90.55 and 90.86 at epoch 165, respectively, but declined to 85.44 and 85.79 at epoch 200, reflecting decreases of 5.11 and 5.07 points compared to their peaks. This suggests that setting the threshold too low can lead to excessive acceptance of pseudo-labels, causing noise accumulation in later training stages and resulting in unstable performance.

By contrast, the 0.01 threshold achieved an accuracy of 90.62 at epoch 165 and maintained 86.36 at epoch 200, representing a smaller decrease of 4.26 points. This relatively minimal performance drop indicates that the system achieved both high initial accuracy and stable performance throughout the self-training process. These results demonstrate that the super-low-threshold strategy not only increases the quantity of labels but also effectively balances learning signals and noise by maintaining noise at manageable levels while providing sufficient training signals.

When the threshold was set at 0.05 or higher, the magnitude of performance decline over successive iterations was limited, but the overall performance remained low from the outset. For example, at a threshold of 0.05, the system recorded 89.48 at epoch 175 and 87.63 at epoch 200, showing relatively stable results but ultimately lower peak and final performances compared to the 0.01 setting. Thresholds of 0.1 or higher (high-threshold settings) limited the number of pseudo-labels to the point where the benefits of self-training were not fully realized.

It is also noteworthy that, despite the performance declines observed in the later stages for some thresholds (e.g., 0.001 and 0.005), their AP still surpassed that of the high-threshold settings (≥0.05). This suggests that the proposed system possesses structural robustness, allowing it to tolerate a certain level of label noise without experiencing drastic performance collapse.

**Figure 13:**
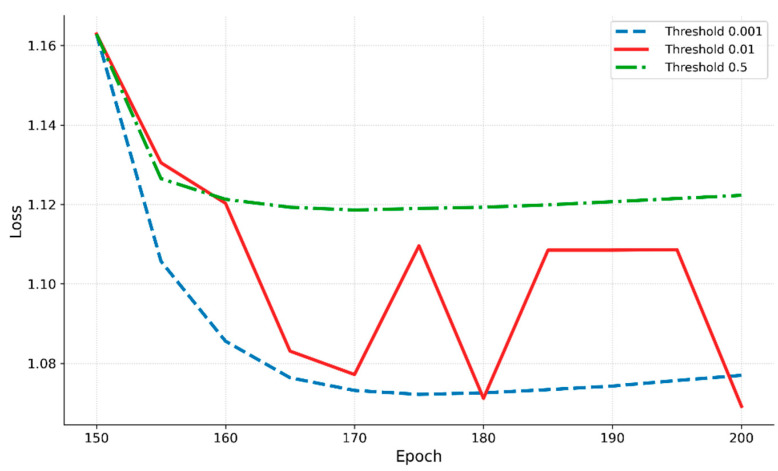
Loss curves during self-training under three pseudo-label confidence thresholds: each line showing 0.001 (blue dashed), 0.01 (red solid), and 0.5 (green dashed–dotted) threshold. The 0.01 threshold demonstrates stable training with minimal loss fluctuation, while 0.001 exhibits late-stage overfitting, and 0.5 indicates underfitting due to limited pseudo-labels.

These observations are further supported by the loss curves presented in Figure 13. The curve corresponding to the 0.01 threshold exhibits fluctuations between epochs 175 and 195, yet the loss remains within a narrow range of approximately 1.08–1.12. In contrast, the 0.001 threshold shows a steady decline in loss up to epoch 180, followed by a slight increase, indicating signs of overfitting due to the accumulation of noise in the pseudo-labels. Meanwhile, the 0.5 threshold maintains an almost flat loss curve throughout the training process, suggesting underfitting caused by insufficient pseudo-labels for effective learning. Although the 0.01 curve shows some variation, it minimizes the overfitting observed at 0.001 and the underfitting observed at 0.5, demonstrating a well-balanced training tendency that avoids both extremes.

Overall, the 0.01 threshold demonstrated the best results in terms of peak performance, sustained accuracy across iterative training, and tolerance to label noise. Therefore, this value was adopted as the baseline for the super-low-threshold strategy in this study.

### 6.5. Validation and Generalizability Across Models and Scenarios

The experimental results strongly support the validity and generalizability of the proposed approach across various object detection models and deployment scenarios. As shown in Table 6, Table 7, Table 8 and Table 9, the proposed system consistently improved accuracy across YOLOv6, YOLOv7, and YOLOv8. Additional results using YOLOv5 (Appendix A) showed similar improvements, confirming that the method generalizes beyond the originally targeted architectures. This consistency across models highlights the approach’s architecture-agnostic nature and implementation flexibility.

The method also showed strong adaptability to domain shift conditions. In camera viewpoint adaptation experiments (Table 10), the adapted models achieved over 90% AP in both top-view → tilted-view and the reverse direction, while source-only models performed substantially worse (36.86 and 69.91 AP, respectively). These results demonstrate that the proposed system generalizes effectively across viewpoint shifts, suggesting robustness to diverse environmental conditions in real-world farms.

In addition, ablation studies, as shown in Table 11, Table 12, Table 13, Table 14 and Table 15, quantify the contribution of each core component. High-entropy frame selection for SLOT (Table 11) and minimal target annotation (Table 12) significantly improved Source-Only baselines’ performance. Early stopping in DAS (Table 13) enhanced the training efficiency, and key frame selection in self-training (Table 14) led to better pseudo-label quality. Table 15 shows the effect of additional transformation strategies, revealing that careful selection of augmentations is essential for optimal performance.

Table 16 further supports the rationale for entropy-based frame selection. It shows that higher-entropy images tend to contain more detectable pigs, justifying the use of entropy as a proxy for informative content in the absence of ground truth.

Finally, Figure 11 validates the reliability of the evaluation procedure. The strong correlation between AP scores on real and synthetic evaluation sets (Figure 11a,b) confirms that our augmented test set accurately reflects actual model performance.

In summary, consistent gains across models, domain conditions, and component settings confirm the repeatability and generalizability of the proposed method. These findings support its scalability and effectiveness in diverse and practical farm environments.

## 7. Limitations and Future Work

The proposed system effectively addresses the domain shift problem and demonstrates robust performance; however, several limitations remain. First, the experiments in this study were conducted solely in five specific pig farm environments. While these environments adequately reflect real farm settings, it is difficult to claim that they fully represent the diversity of all commercial farming environments. Second, the study assumed relatively static camera installations and did not evaluate the system under dynamic conditions where cameras are mobile or the background undergoes rapid changes. In such settings, object positions, background configurations, and lighting conditions can fluctuate significantly over time, potentially causing a frame-based static learning model to produce false positives or experience performance degradation. Notably, when cameras are in motion, the same object may appear at varying sizes or against different backgrounds across frames, further intensifying the decline in accuracy. Third, a copy-and-paste-based synthetic dataset was employed for fitness evaluation within the DAS. However, this approach may not fully capture key factors observed in real farm environments, such as background consistency and occlusion, possibly leading to visually unnatural results and reducing the representativeness of the evaluation data. Finally, the system’s effective domain adaptation depends on augmentation parameter searches and iterative self-training procedures, which require substantial computational resources. As additional adaptation becomes necessary over time, the operational burden increases, introducing constraints to the scalability and applicability of the system.

To overcome these limitations, future research will proceed in the following directions. First, additional experiments will be conducted across farms with various pig breeds, barn structures, and lighting conditions to verify the system’s generalization performance. Second, a self-training strategy incorporating temporal consistency will be introduced to ensure robustness in dynamic environments. For example, a tracking module could assign pseudo-labels only when the same object is consistently detected across consecutive frames. Alternatively, a temporal ensemble approach could be applied to integrate confidence scores from temporally adjacent frames, thereby suppressing transient noise and stabilizing label quality. Thirdly, in building a dataset to be used in the DAS algorithm, a dataset close to a commercial farm environment can be created by applying a data generation algorithm using background and foreground. Moreover, advanced synthetic evaluation techniques, such as GAN-based image generation, may be explored further to narrow the gap between synthetic and real-world data distributions. Lastly, future research will focus on developing a mechanism that can automatically determine the need for model retraining by monitoring domain shift indicators in real time. The mechanism would enable the system to proactively respond to continuous environmental changes while minimizing the need for manual intervention.

These follow-up studies are expected to enhance the system’s adaptability to environmental changes, operational efficiency, and learning stability comprehensively. Ultimately, they may pave the way for developing a practical and sustainable intelligent livestock monitoring system applicable in real farm settings.

## 8. Conclusions

This study presented a novel domain adaptation method for pig detection that combines a genetic-algorithm-optimized data augmentation strategy with a self-training scheme to overcome severe performance degradation caused by domain shifts in real-world farm settings. A key novelty of the approach is using only a SLOT sample from the new environment and DAS to create a robust base model. This base model is progressively improved through a super-low-threshold self-training process that leverages abundant unlabeled target data. The proposed method effectively adapts a pig detection model to new domains with minimal manual effort by integrating these components, minimal supervised input, targeted augmentation, and aggressive self-training into one unified system.

The experimental results demonstrate the effectiveness and practicality of this system. The adapted model’s detection accuracy more than doubled compared to a source-only baseline, rising from roughly 37% to 91%, which is on par with the performance of a fully supervised model in the target domain. The approach also proved robust across various domain shift scenarios. For example, it maintained high accuracy when the camera perspective changed from a top-down view to a tilted angle and under different pen conditions and lighting setups. These outcomes confirm that our method mitigates the domain shift issues common in livestock environments. Notably, the system achieves this improvement while requiring only one new annotated image, highlighting a substantial reduction in the data-labeling burden. The improvement makes the solution highly practical for commercial farm deployment, where obtaining large labeled datasets is often infeasible.

In conclusion, the proposed system offers a powerful and efficient domain adaptation solution. It enables quick, cost-effective model customization to each farm’s conditions and is expected to maintain reliable performance over time in real operational settings. By drastically lowering the barrier to deploying accurate vision models in new environments, our approach can contribute significantly to advancing smart livestock farming and improving the efficiency of animal monitoring systems.

## Figures and Tables

**Table 1:** Summary of recent studies on domain-adaptive pig detection (published between 2019 and 2025). The summarized studies on pig object detection included domain shift results or applied semi-supervised learning.

Training Method	Model	Domain Shift Result	Domain Shift Adaptation	Labeled Target Data Utilization	Reference
Supervised	Fully Convolutional Network	✔	✘	✘	Psota et al., 2019 [52]
Supervised	Faster R-CNN	✔	✘	✘	Riekert et al., 2020 [53]
Supervised	YOLOv4	✔	✔	✘	Zhang et al., 2022 [54]
Supervised	YOLOv5	✔	✘	✘	Liu et al., 2023 [55]
Supervised	Anchor-Free Center-Based (AFCB)	✔	✔	✘	Mattina et al., 2023 [56]
Supervised	IO-YOLOv5	✔	✔	✘	Lai et al., 2023 [57]
Semi-supervised	YOLOv8	✔	✔	✘	Wutke et al., 2024 [58]
Semi-supervised	YOLOv7 + Cycle GAN	✔	✔	✔	Wang et al., 2025 [59]
Semi-supervised	YOLOv8	✔	✔	✔	Proposed

✔: Applied or included, ✘: not applied

**Table 2:** Summarizing the search space to be explored for data augmentation in the DAS.

Augmentation Parameter	Definition
$N_{image}$	Number of augmented images
$P_{zoom\_in}$	Probability of zoom-in
$M_{zoom\_in}$	Magnitude of zoom-in
$P_{zoom\_out}$	Probability of zoom-out
$M_{zoom\_out}$	Magnitude of zoom-out
$F_{trans}$ (0: original, 1: translateX, 2: translateY, 3: rotate, 4: shear, 5: flipH, 6: flipV)	Image transformation flag
$P_{trans}$	Probability of image transformation
$M_{trans}$	Magnitude of image transformation

**Table 3:** The specifications of the source and target data used for the model’s training and testing. The Hadong data refer to the images extracted through key frame selection.

Name	Domain	Number of Pigs	Resolution	Camera Angle	Train/ Test	Label Availability	Frames	Example Image
Hamyang	Source	21	1200 × 600	Top-View	Train	Yes	342	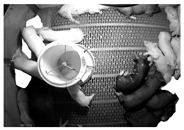
Jochiwon	Source	23	512 × 512	Top-View	Train	Yes	917	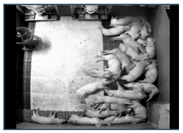
Chungbuk	Source	5 or 9	1200 × 600	Top-View	Train	Yes	1182	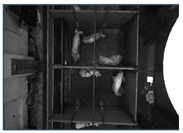
AI Hub [75]	Source	Variable	1920 × 1080	Top-View	Train	Yes	3520	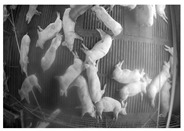
Hadong	Target	Variable	1920 × 1080	Tilted-View	Train	Yes	1 (SLOT)	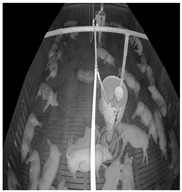
					Train	No	3999	
					Test	Yes	1024	

**Table 4:** Summary of the data augmentation search space to be explored in the DAS.

Augmentation Parameter	Range
$N_{image}$	10–500
$P_{zoom\_in}$	0–0.5 [0, 0.1, 0.2, 0.3, 0.4, 0.5]
$M_{zoom\_in}$	1.2–1.9 [1.2, 1.3, 1.4, 1.5, 1.6, 1.7, 1.8, 1.9]
$P_{zoom\_out}$	0–0.5 [0, 0.1, 0.2, 0.3, 0.4, 0.5]
$M_{zoom\_out}$	0.2–0.9 [0.2, 0.3, 0.4, 0.5, 0.6, 0.7, 0.8, 0.9]
$F_{trans}$ [0: original, 1: translateX, 2: translateY, 3: rotate, 4: shear, 5: flipH, 6: flipV]	0–6 [0, 1, 2, 3, 4, 5, 6]
$P_{trans}$	0.1–1 [0.1, 0.2, 0.3, 0.4, 0.5, 0.6, 0.7, 0.8, 0.9, 1.0]
$M_{trans}$	1–8 [1, 2, 3, 4, 5, 6, 7, 8]

**Table 5:** DAS results selected parameters. This table shows two parameters with the highest AP and two parameters with the lowest AP among the 60 chromosomes. The AP was evaluated using YOLOv8 and evaluation data generated by the copy-and-paste technique.

Augmentation Parameter	TOP1	TOP2	TOP59	TOP60
$N_{image}$	48	140	337	388
$P_{zoom\_in}$	0	5	3	4
$M_{zoom\_in}$	1	2	1	7
$P_{zoom\_out}$	5	4	1	1
$M_{zoom\_out}$	8	8	7	9
$F_{trans}$	0	3	6	3
$P_{trans}$	1	6	8	7
$M_{trans}$	1	2	1	9
AP (%)	48.30	45.37	30.94	18.63

**Table 6:** Comparison of base models trained for 150 epochs. Oracle refers to the results of the supervised learning. Source-Only refers to the results where target data were not involved in the training. SLOT refers to the results where SLOT data were added to source-only training data. DAS refers to the results where data generated through DAS were additionally included in the training data used for the SLOT method. Training duration reflects the approximate time required to train each model using the DAS method.

Model	Params (M)	FLOPs (G)	Training Duration (hours)	Method	Number of Target Labels	AP (%)
YOLOv6	18.51	45.20	8.50	Oracle	4000	88.01
				Source-Only	0	61.67
				SLOT	1	81.99
				DAS	1 (SLOT) + 48 (augmented)	89.67
YOLOv7	36.91	104.50	10.94	Oracle	4000	89.69
				Source-Only	0	53.52
				SLOT	1	87.83
				DAS	1 (SLOT) + 48 (augmented)	89.60
YOLOv8	25.90	79.32	7.12	Oracle	4000	95.15
				Source-Only	0	36.86
				SLOT	1	80.25
				DAS	1 (SLOT) + 48 (augmented)	85.27

**Table 7:** A comparison table of the self-training and DD results (%) for each model. AP$\tau_{conf}$ refers to the results where data with a threshold of $\tau_{conf}$ or higher was designated as pseudo-label data. Bold values indicate the highest accuracy for each confidence score threshold, and underlined values indicate the highest accuracy within each model for self-training and DD.

Model	Epoch	Self-Training					DD				
		AP_0.01_	AP_0.05_	AP_0.1_	AP_0.3_	AP_0.5_	AP_0.01_	AP_0.05_	AP_0.1_	AP_0.3_	AP_0.5_
YOLOv6	150	89.67 (DAS)									
	155	90.09	90.06	90.06	90.08	90.06	90.04	90.09	90.06	90.06	90.06
	160	90.39	90.40	90.40	90.38	90.40	90.40	90.38	90.39	90.40	90.40
	165	90.43	90.48	90.48	90.44	90.48	90.45	90.44	90.46	90.48	90.48
	170	90.48	90.49	90.49	90.48	90.49	90.49	90.49	90.49	90.49	90.49
	175	**90.50**	**90.57**	**90.57**	**90.54**	**90.57**	**90.57**	**90.52**	**90.53**	**90.57**	**90.57**
YOLOv7	150	89.60 (DAS)									
	155	90.16	90.10	90.06	90.12	90.23	91.25	91.16	91.12	91.17	91.26
	160	90.38	90.34	90.22	90.35	90.49	91.87	91.74	91.75	92.02	91.87
	165	90.48	90.44	90.49	90.48	90.72	92.50	92.30	92.26	92.62	92.42
	170	90.48	90.69	90.53	90.67	90.85	92.80	92.83	92.55	93.21	92.86
	175	**90.63**	**90.70**	**90.64**	**90.72**	**90.98**	**93.11**	**93.31**	**93.00**	**93.70**	**93.25**
YOLOv8	150	85.27 (DAS)									
	155	87.28	86.54	86.38	85.94	**85.42**	87.80	87.50	87.39	86.93	86.32
	160	88.29	87.29	86.87	86.16	85.37	89.86	88.43	88.17	87.36	86.38
	165	89.53	87.81	87.35	86.22	85.38	**90.62**	89.04	88.52	87.52	86.43
	170	90.03	88.23	87.70	86.30	85.36	90.45	**89.48**	88.63	87.63	**86.47**
	175	**90.07**	**88.52**	**87.95**	**86.37**	85.39	89.85	**89.48**	**88.73**	**87.64**	86.46

**Table 8:** A summary of the experiments conducted based on the YOLOv8 model.

SLOT	DAS	DD	Super-Low Threshold	AP (%)
				36.86
✔				80.25
✔	✔			85.27
✔	✔	✔		86.47
✔	✔	✔	✔	90.62

✔: The check mark indicates whether the corresponding method is activated.

**Table 9:** A comparison table of the AP results (%) based on different self-training techniques.

Method	YOLOv6	YOLOv7	YOLOv8
Naive Self-Training [67]	80.56	64.59	62.04
DD [69]	79.30	75.98	65.29
Proposed System	90.57	93.11	90.62

**Table 10:** Comparison of the AP results (%) for the proposed method based on changes in the camera’s perspective.

Camera Angle (Source → Target)	Source-Only	SLOT	DAS	Proposed System
Top-View → Tilted-View	36.86	80.25	85.27	90.62
Tilted-View → Top-View	69.91	83.80	86.90	90.16

**Table 11:** Base model training results for SLOT data selected based on entropy values. The “Highest” method shows the results of training the model using the image with the highest entropy value among the extracted target-domain data. The “Lowest” method shows the results of training the model using the image with the lowest entropy value among the extracted target-domain data.

Method	Entropy	AP (%)
Highest Entropy	0.822795	85.27
Lowest Entropy	0.797567	80.43

**Table 12:** AP evaluation results based on the number of SLOT data samples. When the number of SLOT data samples is 0, the model was trained using only source data.

Number of SLOT Data Samples	AP (%)
0 (Source-Only)	36.86
1	85.81
5	88.62
10	92.48
100	93.67
1000	95.26
4000	95.78

**Table 13:** A comparison of the results based on whether early stopping was applied during the DAS process.

Early Stop	G	C	Total Epochs	Operating Time (hours)	AP (%)
	3	60	1800	103	77.35
✔			1595	86	85.27

✔: The check mark indicates whether the corresponding method was activated.

**Table 14:** A comparison table of model AP (%) based on whether the key frame selection algorithm was applied. In APep, ep represents the number of epochs for the model. Bold values indicate the higher accuracy between the two methods for each epoch.

AP_ep_	Key Frame Selection Algorithm	
		✔
AP_150_	85.27	
AP_155_	**88.04**	87.80
AP_160_	**89.95**	89.86
AP_165_	90.29	**90.62**
AP_170_	90.01	**90.45**
AP_175_	89.53	**89.85**

✔: The check mark indicates whether the corresponding method was activated.

**Table 15:** AP (%) comparison on the application of image transformation techniques.

Proposed Method	Perspective Transformation [81]	DOG [82]	AP
**✔**			90.62
**✔**	**✔**		90.73
**✔**	**✔**	**✔**	79.82

✔: The check mark indicates whether the corresponding method was activated.

**Table 16:** The difference in the average number of objects between the high-entropy and low-entropy groups was analyzed. The entropy groups indicate the number of images selected for each category, while the average object count represents the mean number of objects present in the images within the upper- and lower-entropy groups.

Entropy Group Category	High-Entropy Group Average Number of Objects	Low-Entropy Group Average Number of Objects
A (10 frames)	26.5	19.7
B (100 frames)	26.34	20.16
C (1000 frames)	24.28	20.76

**Table 17:** Comparison of AP (%) changes across self-training iterations (epochs 155–200) according to confidence threshold values. AP $\tau_{conf}$ refers to the results where data with a threshold of $\tau_{conf}$ or higher was designated as pseudo-label data. Bold values indicate the highest accuracy within each threshold, and underlined value indicate the highest accuracy across all thresholds.

Epoch	AP_0.001_	AP_0.005_	AP_0.01_	AP_0.05_	AP_0.1_	AP_0.3_	AP_0.5_
155	88.38	88.02	87.80	87.50	87.39	86.93	86.32
160	90.47	90.16	89.86	88.43	88.17	87.36	86.38
165	**90.55**	**90.86**	**90.62**	89.04	88.52	87.52	86.43
170	89.73	90.41	90.45	**89.48**	88.63	87.63	**86.47**
175	88.68	89.51	89.85	**89.48**	88.73	**87.64**	86.46
180	87.77	88.51	89.09	89.22	**88.76**	87.58	86.43
185	87.06	87.61	88.32	88.90	**88.76**	87.47	86.37
190	86.43	86.85	87.59	88.44	88.74	87.40	86.31
195	85.91	86.26	86.95	87.94	88.52	87.33	86.21
200	85.44	85.79	86.36	87.34	88.26	87.20	86.15

## Data Availability

The original contributions presented in this study are included in the article. Further inquiries can be directed to the corresponding authors.

## Appendix A. Performance Verification with YOLOv5

This appendix evaluates the performance of the proposed system using the YOLOv5 model to verify its applicability across different object detection models, particularly in the context of DAS and self-training.

DAS is performed based on the YOLOv5 model. Table A1 presents the results of applying DAS using both YOLOv5 and YOLOv8. The augmentation parameters shown represent the highest AP for each model. As with YOLOv8, the YOLOv5 results also demonstrated superior performance when the number of images was low. In the case of zoom operations, only zoom-in was applied for YOLOv5, whereas zoom-out was used for YOLOv8, indicating that zoom-in and zoom-out can serve as interchangeable augmentation strategies. A value of 0 (i.e., original) was selected for both YOLOv5 and YOLOv8, suggesting that the highest AP was achieved when no additional augmentation was applied to individual objects.

**Table A1:** The results of applying the DAS with YOLOv5 and YOLOv8 models are compared. The table shows the parameters that achieved the highest AP among the total of 60 chromosomes.

Augmentation Parameter	YOLOv5	YOLOv8
$N_{image}$	24	48
$P_{zoom\_in}$	4	0
$M_{zoom\_in}$	2	1
$P_{zoom\_out}$	0	5
$M_{zoom\_out}$	1	8
$F_{trans}$	0	0
$P_{trans}$	1	1
$M_{trans}$	1	1
*AP (%)*	56.46	48.30

Table A2 compares the training results for the base models, used as initial models in the self-training process, based on different training strategies and various YOLO series models. In this experiment, DAS was conducted using the YOLOv5 model as a baseline.

**Table A2:** These are the results of training YOLOv5, YOLOv6, and YOLOv7 models using data generated by applying the DAS based on YOLOv5. The training duration reflects the approximate time required to train each model using the DAS method.

Epochs	Model	Params (M)	FLOPs (G)	Training Duration (hours)	Method	AP (%)
150	YOLOv5	20.86	47.87	5.35	Oracle	93.07
					Source-Only	59.02
					SLOT	86.15
					DAS	88.80
	YOLOv6	18.51	45.20	8.50	Oracle	88.01
					Source-Only	61.67
					SLOT	81.99
					DAS	88.55
	YOLOv7	36.91	104.50	10.94	Oracle	89.69
					Source-Only	53.52
					SLOT	87.83
					DAS	89.14

The results show that incorporating DAS-augmented data into the training process of YOLOv5, YOLOv6, and YOLOv7 led to higher AP values than the SLOT method. Specifically, YOLOv5 achieved up to a 2.65-point improvement over SLOT, while YOLOv6 and YOLOv7 recorded gains of 2.81 and 2.60 points, respectively. Overall, DAS improved AP across all three models, with YOLOv7 achieving the highest AP of 89.14.

These findings demonstrate that DAS is applicable across various model architectures and can contribute to performance enhancement regardless of the underlying model structure.

Table A3 presents the results of applying self-training and DD to the base model trained with DAS-augmented data based on the YOLOv5 architecture. Similar to the findings based on the YOLOv8 model, the experiments using YOLOv5 also demonstrated that applying a low confidence threshold improved accuracy.

**Table A3:** A table comparing the results (%) of self-training and DD based on a model trained for 150 epochs with data generated through DAS, using the YOLOv5 model as the foundation. AP $\tau_{conf}$ refers to the results where data with a threshold of $\tau_{conf}$ or higher were designated as pseudo-label data. Bold values indicate the highest accuracy for each confidence score threshold, and underlined values indicate the highest accuracy within each model for self-training and DD.

Model	Epoch	Self-Training					DD				
		AP_0.01_	AP_0.05_	AP_0.1_	AP_0.3_	AP_0.5_	AP_0.01_	AP_0.05_	AP_0.1_	AP_0.3_	AP_0.5_
YOLOv5	150	88.80 (DAS)									
	155	90.64	90.20	89.85	**89.12**	**88.67**	91.70	91.46	91.28	90.65	90.17
	160	91.44	90.89	90.27	89.05	88.25	**92.40**	92.18	91.76	90.95	90.22
	165	91.93	91.27	90.58	88.95	87.93	92.18	**92.52**	91.98	91.08	90.20
	170	92.09	91.47	90.75	88.90	87.71	91.42	92.46	**92.07**	91.26	90.25
	175	**92.16**	**91.60**	**90.85**	88.86	87.53	90.27	92.31	**92.07**	**91.41**	**90.26**
YOLOv6	150	88.55 (DAS)									
	155	89.00	89.02	89.03	89.03	89.03	89.05	89.03	89.04	89.04	89.04
	160	89.23	89.22	89.31	89.26	89.26	89.27	89.26	89.22	89.22	89.22
	165	89.27	89.32	89.41	89.32	89.32	89.34	89.28	89.33	89.33	89.33
	170	89.34	89.37	89.45	89.35	89.35	89.41	89.33	89.40	89.40	89.40
	175	**89.35**	**89.40**	**89.47**	**89.40**	**89.40**	**89.45**	**89.38**	**89.44**	**89.44**	**89.44**
YOLOv7	150	89.14 (DAS)									
	155	89.96	89.91	89.74	89.86	89.71	90.93	90.75	90.82	90.66	90.51
	160	90.04	90.03	89.98	89.95	89.93	91.68	91.66	91.56	91.46	91.45
	165	90.15	90.22	90.07	90.04	90.06	92.01	92.01	92.03	91.80	91.81
	170	90.20	90.29	90.12	90.12	90.19	92.28	92.22	92.21	91.96	**91.89**
	175	**90.26**	**90.44**	**90.13**	**90.18**	**90.29**	**92.42**	**92.57**	**92.40**	**91.97**	**91.89**

This effect was particularly notable in the self-training process of the YOLOv5 model; prior to applying the low confidence threshold, the model achieved an AP of 88.67, which increased to 92.16 after its application—representing a performance gain of 3.49. These results are consistent with those observed in the experiments using YOLOv8, reinforcing the effectiveness of a super-low-threshold strategy in enhancing model performance across different architectures.
